# Antioxidant Effect of a *Fucus vesiculosus* Extract on Intestinal Ischemia/Reperfusion Injury in Rats: A Biochemical and Histological Study

**DOI:** 10.3390/antiox14060624

**Published:** 2025-05-23

**Authors:** Desirée Sánchez-Bonet, Carolina Padrón-Sanz, José Miguel Lloris-Cejalvo, José Miguel Lloris-Carsí, Dolores Cejalvo-Lapeña

**Affiliations:** 1 Escuela de Doctorado, Universidad Católica de Valencia “San Vicente Mártir”, Plaza de San Agustín, 3, 46001 Valencia, Spain; desanbo@gmail.com (D.S.-B.); jmllorisc@gmail.com (J.M.L.-C.); 2 Centro de Investigación Traslacional San Alberto Magno, Universidad Católica de Valencia “San Vicente Mártir”, C/Quevedo, 2, 46001 Valencia, Spain; dolores.cejalvo@ucv.es; 3 Facultad de Medicina, Departamento de Cirugía, Universidad de Valencia, Avda. Blasco Ibáñez, 13, 46010 Valencia, Spain; jm_lloris@mac.com

**Keywords:** *Fucus vesiculosus*, antioxidant, polyphenols, intestinal ischemia/reperfusion

## Abstract

*Fucus vesiculosus* is a brown seaweed known for its strong antioxidant properties, mainly attributed to its high polyphenolic content. This study aimed to evaluate the antioxidant protective effect of an optimised *F. vesiculosus* extract in an experimental model of intestinal ischemia/reperfusion (I/R) injury, considering the intestine as particularly vulnerable to this pathology. Seventy-two male Wistar albino rats were randomly divided into twelve groups: Sham, I/R groups (3 and 24 h reperfusion), I/R plus vehicle groups (three application times, 3 h reperfusion), and I/R plus *F. vesiculosus* extract groups (three application times, 3 and 24 h reperfusion). Intestinal injury was assessed through biochemical markers (malondialdehyde [MDA], superoxide dismutase [SOD], catalase [CAT], glutathione peroxidase [GPx], and mieloperoxidase [MPO]), inflammatory cytokines (interleukin 1 β [IL-1β] and interleukin [IL-10]), and histological analysis. Results demonstrated that treatment with *F. vesiculosus* significantly reduced oxidative stress and inflammation caused by I/R injury (*p* < 0.05), restoring analysed parameters (MDA, SOD, CAT, IL-10) to levels comparable to the Sham group. Histological examination confirmed the preservation of intestinal mucosal integrity following *F. vesiculosus* administration. These findings suggest that the antioxidant extract from *F. vesiculosus* effectively protects against intestinal I/R injury, highlighting its potential for clinical use in preventing and managing this pathological condition, particularly in surgical contexts.

## 1. Introduction

Molecular oxygen is essential for all aerobic organisms, but environmental conditions and pollutants can disrupt the functioning of electron transport chains and lead to the formation of oxygen free radicals (ROS) [1]. ROS are highly reactive molecules that can cause significant damage to cellular components including lipids, proteins and nucleic acids [2,3]. Under normal conditions, organisms are able to maintain stable ROS levels through endogenous antioxidant mechanisms [4]. These antioxidant systems are composed of enzymes such as superoxide dismutase (SOD), catalase (CAT) and glutathione peroxidase (GPx). However, when an imbalance occurs between ROS production and the organism’s capacity to neutralise them, a biological phenomenon known as oxidative stress occurs [1,2,5,6].

There are several pathophysiological processes in which oxidative stress plays an important role, ischemia/reperfusion (I/R) being one of them [7,8]. During the ischemic phase, the lack of oxygen and nutrients causes a shift in cellular metabolism to anaerobic pathways. This shift results in a loss of cell function that can lead to cell death [7,9]. In the second phase (reperfusion), the restoration of blood flow could be seen as a recovery process, but paradoxically, the opposite effect occurs. The sudden increase in oxygen availability leads to an overproduction of free radicals, like superoxide (O_2_^−^) and hydrogen peroxide (H_2_O_2_), both presenting a high potential to damage cellular structures [8,10,11].

The small intestine is recognised as one of the most vulnerable organs to this type of injury, with one of the highest morbidity and mortality rates [12]. Intestinal I/R injury is associated with numerous acute pathological conditions, including small bowel transplantation, cardiopulmonary resuscitation, septic shock and mesenteric artery embolisation [13]. This phenomenon is usually caused by obstruction of arterial mesenteric vessels, although in rare cases, it can also involve venous vessels, as a result of thrombi or emboli [14]. Oxidative stress occurring during an intestinal I/R process can trigger the destruction of the intestinal mucosal barrier, increase vascular permeability, promote bacterial translocation, and trigger the release of inflammatory mediators. In addition, it can cause a cellular response involving apoptosis, autophagy and necroptosis [15,16].

Antioxidants are considered an essential resource to control the overproduction of ROS and prevent oxidative damage, as they are able to neutralise free radicals in the body’s cells, producing a more stable and less harmful radical [17]. Currently, there is an increasing interest in obtaining new antioxidants from natural sources, as they are considered safer and more effective treatments to combat diseases associated with oxidative damage [18]. In this context, numerous studies have focused on marine-derived products, especially seaweeds, which have gained relevance due to their ability to synthesise a wide variety of secondary metabolites [17,19]. The production of this wide variety of bioactive compounds is attributed to the extreme environmental conditions to which these organisms have adapted, especially those algae that grow in intertidal zones, where they are exposed to fluctuating ocean levels that produce constant and alternating changes in high and low salinity, low or high temperature, high pressure, low nutrient availability and low or high exposure to sunlight [20,21]. These adverse conditions favour the formation of ROS and other oxidative agents [22,23]; however, the absence of oxidative damage to membrane lipids suggests that algal cells have developed a complex defence system based on potent antioxidant compounds [21,24].

Among the bioactive compounds found in marine algae, polyphenols have gained significant interest due to their high antioxidant potential [25]. The chemical configuration of these compounds enables them to scavenge free radicals through their hydroxyl groups [26], making their mechanism of action primarily centred on ROS scavenging, inhibition of lipid peroxidation, and activation of the endogenous antioxidant system [26,27,28,29,30].

Numerous studies have shown that brown algae (Phaeophyceae) present higher antioxidant potential compared to green (Chlorophyta) and red (Rhodophyta) algae [22,31,32,33,34]. In addition, a higher concentration of polyphenols has been observed in this group, highlighting florotannins, a type of polyphenol with potent antioxidant properties that are exclusively synthesised by brown algae [26,32,35,36,37]. Within the brown algae, the order Fucales stands out for its higher antioxidant potential [32,38]. In particular, *Fucus vesiculosus* has attracted considerable attention in recent years due to its remarkable bioactivity, outperforming other species in comparative studies [32,39,40], as well as for its richness in bioactive compounds, such as polyphenols [32].

Therefore, the aim of this study is to determine the efficacy of polyphenols extracted from the algae *Fucus vesiculosus* on intestinal ischemia/reperfusion injury using an animal experimental model.

## 2. Materials and Methods

### 2.1. Seaweed Pre-Treatment

*Fucus vesiculosus* seaweed was purchased by the pharmaceutical company FAGRON (Terrassa, Barcelona) already pre-treated: dehydrated and ground into a homogeneous powder in a hermetically sealed container with a capacity of 1 kg.

### 2.2. Seaweed Extracts Preparation

In an Erlenmeyer flask, 5 g of algae sample was mixed with 30 mL of 70% acetone. The mixture was then subjected to an extraction process using an orbital incubator (Sartorius Stedim Biotech GmbH, Göttingen, Germany) at a temperature of 25 °C for 60 min. After this time, the samples were centrifuged at 2000× *g* for 20 min (Thermo Scientific Heraeus, Waltham, USA), and the supernatants were filtered using 0.45 μm nylon filters. Before its administration to the rats, the extract was rotary evaporated (Büchi Labortechnik AG, Flawil, Switzerland) to remove all acetone, then resuspended in 10 mL of 10% ethanol (*v/v*). The smallest amount of alcohol possible was used, but enough to ensure resuspension of the entire dried extract.

### 2.3. Characterisation of Polyphenols and Determination of the Extract’s Antioxidant Activity

The Folin–Ciocalteu method was used to determine the total polyphenol content in accordance with Julkunen-Tiito [41]. Antioxidant activity was measured using the 2,2-diphenyl-1-picrylhydrazyl free radical scavenging activity (DPPH-RSA) method described by Chu et al. [42] with some modifications. Chromatographic analyses for the description of the polyphenolic profile were performed using a method adapted from López et al. [43] with a reverse-phase high-performance liquid chromatography (RP-HPLC) equipment coupled to a diode array detector (DAD). The detailed procedural descriptions of these methodologies have been published in a previous study in which the extraction of the alga *F. vesiculosus* was optimised [44].

### 2.4. Animals

Male Wistar albino rats (*n* = 72) weighing between 200 and 300 g were used in the present study. The animals underwent a 7-day acclimatisation period in a controlled environment with temperatures ranging from 22 to 24 °C and humidity levels between 50 and 70%. They had unrestricted access to food and water. The care and treatment of the rats were conducted in compliance with the animal welfare guidelines outlined in the Guide for the Care and Use of Laboratory Animals, following European Union regulations. The animal experimentation procedure described in this study was approved by the Generalitat Valenciana (General Directorate of Agricultural Production and Livestock), in accordance with current animal welfare regulations (Royal Decree 53/2013).

### 2.5. Experimental Groups

The impact of *F. vesiculosus* extract on I/R injury was assessed in two phases. The first phase involved 60 min of ischemia followed by 3 h of reperfusion to evaluate the effect of the extract on early injury events, such as oxidative stress and inflammation. The second phase, with 60 min of ischemia followed by 24 h of reperfusion, allowed for the evaluation of injury progression, systemic inflammatory responses, and tissue repair processes. On the other hand, the antioxidant extract was administered at three different time points: t.1 (24 h before ischemia), to evaluate its potential protective or preventive effect; t.2 (at the onset of ischemia), to assess whether the extract can act on the damage initiated during the hypoxic phase; and t.3 (at the initiation of reperfusion), to evaluate its reparative capacity during the reperfusion phase.

The excipient group allowed us to verify that the substance used to resuspend the seaweed extract (10% ethanol) had no effect on the lesion produced by IR and to verify that the effects produced are only due to the seaweed extract. To reduce the number of animals used, the excipient and sham groups were not studied in the second phase once it was determined that their conditions did not influence the I/R injury.

This study involved twelve groups of animals (*n* = 6 animals per group), distributed as follows (Table 1):

### 2.6. Surgical Procedure

Following an overnight fast, animals were anesthetised with ketamine 80 mg/kg (Merial Laboratory, Barcelona, Spain) and xylazine 10 mg/kg (Calier Laboratory, Barcelona, Spain) via intraperitoneal injection. The rats were allowed to breathe on their own during the procedure. A heating lamp was employed to maintain their body temperature at approximately 37 °C. The abdominal area was shaved and disinfected with Betadine^®^ prior to aseptic surgery. After performing a midline laparotomy, the superior mesenteric artery (SMA) was carefully exposed and occluded just below the aorta, resulting in collateral interruption, for 60 min using atraumatic microvascular clamps (Scanlan International, St. Paul, MN, USA). Following 60 min of ischemia, the clamp was taken out, and reperfusion was initiated.

For animals subjected to I/R for 24 h, the abdominal incisions were sealed using continuous 2/0 silk sutures (B. Braun Surgical SA, Rubí, Spain). All rats were anesthetised and euthanised 24 h after reperfusion. Tissue samples were taken at 3 h or 24 h of reperfusion to assess the intestinal damage caused by I/R. Segments of jejunum (2 cm from the third intestinal loop) were excised, frozen using liquid nitrogen, and stored at –80 °C for subsequent biochemical analysis of lipoperoxidation (measured by malondialdehyde [MDA] levels), enzymatic activity of superoxide dismutase (SOD), catalase (CAT), glutathione peroxidase (GPx) and myeloperoxidase (MPO), as well as the quantification of inflammation markers (Il-1β and IL-10).

### 2.7. Intestinal Samples Pre-Treatment

The selected bowel segment was placed into a tube containing phosphate-buffered saline (PBS) as a washing buffer, along with protease inhibitors to prevent degradation of the target proteins. The next step involved mechanically homogenising the tissue sample for 1 min using an Ultraturrax™ (IKA T10 basic) at maximum speed. This procedure was performed at a low temperature to minimise protein degradation. To break down cell membranes, 20 µL of Triton X-100 (Sigma-Aldrich, St. Louis, MO, USA) was added. Lastly, the samples were centrifuged at 10,000× *g* for 10 min at 4 °C, and the supernatant was stored at −80 °C for future analysis.

### 2.8. Biochemical Analysis

#### 2.8.1. Malondialdehyde (MDA) Activity Assay

The assay method used was based on the procedure carried out by Ozkan et al. [45]. Firstly, 50 µL of tissue sample, 150 µL of 60% perchloric acid (Fisher Scientific UK Ltd., Loughborough, UK) and 150 µL of thiobarbituric acid (Merck KGaA, Darmstadt, Germany) were mixed and incubated at 95 °C for 55 min with continuous agitation at 300 rpm. Afterwards, the mixture was cooled to −20 °C for 10 min. Then, 100 µL of 20% trichloroacetic acid (Merck KGaA, Darmstadt, Germany) and 250 µL of *n*-butanol (Acros Organics, Geel, Belgium) were added to the vials. The mixture was vortexed and centrifuged at 13,000× *g* for 6 min. Finally, the absorbance of the butanol layer was measured at 532 nm using a Nanodrop 2000c (Thermo Fisher Scientific, Waltham, MA, USA). MDA concentration was determined from a standard calibration curve generated using 1,1,3,3-tetraethoxypropane (TEP). The results were expressed in nmol per mg of protein.

#### 2.8.2. Measurement of Superoxide Dismutase (SOD) Activity

SOD activity was assessed using a commercial kit (Superoxide Dismutase Assay Kit, Cayman Chemical Company, Ann Arbor, MI, USA), which utilises a tetrazolium salt to detect superoxide radicals generated by xanthine oxidase and hypoxanthine. In the assay, one unit of SOD is set as the amount of enzyme required to produce 50% of the superoxide radical dismutation, which is measured by the change in absorbance at a wavelength of 450 nm. Victor X5 Microplate Reader (PerkinElmer, Waltham, MA, USA) was used to determine absorbance values, and final results were expressed in U/mg.

#### 2.8.3. Measurement of Catalase (CAT) Activity

CAT activity in the tissue was measured using a commercial assay kit (Catalase Assay Kit, Cayman Chemical Company, Ann Arbor, MI, USA). The method relies on the enzyme’s reaction with methanol in the presence of an optimal concentration of H_2_O_2_. The resulting formaldehyde was quantified colourimetrically in the tissue homogenate at a wavelength of 540 nm with a Victor X5 Microplate Reader (PerkinElmer, Waltham, MA, USA). The results were given in nmol/min/mg.

#### 2.8.4. Measurement of Glutathione Peroxidase (GPx) Activity

GPx activity was indirectly measured using a commercial assay kit (Glutathione peroxidase Assay Kit, Cayman Chemical Company, Ann Arbor, MI, USA), which involves a reaction coupled with glutathione reductase. The oxidised glutathione formed during the reduction of hydroperoxides by the enzyme is converted back to its reduced form by GPx and nicotinamide adenine dinucleotide phosphate (NADPH). The decrease in NADPH concentration is monitored by measuring lower absorbance at 340 nm with a Victor X5 Microplate Reader (PerkinElmer, Waltham, MA, USA). Final results were expressed in nmol/min/mg.

#### 2.8.5. Mieloperoxidase (MPO) Activity Assay

For the analysis of MPO in intestinal tissue, a rat ELISA kit (Hycult Biotech, Uden, The Netherlands), based on a sandwich solid phase enzyme-linked immunosorbent assay, was used. The MPO level was determined by measuring the colour change at a wavelength of 450 nm using a Victor X5 Microplate Reader (PerkinElmer, Waltham, MA, USA). The results were expressed as ng/mg prot.

#### 2.8.6. Determination of Interleukins Il-1β and Il-10

The commercial kits Quantikine^®^ ELISA Rat IL-1β Immunoassay and Quantikine^®^ ELISA Rat IL-10 Immunoassay (R&D Systems, Minneapolis, MN, USA) were used to determine intestinal tissue inflammation based on IL-1β and Il-10 levels. The assays are based on the quantification of Rat IL-1β and IL-10 in cell and tissue lysates using the sandwich-type quantitative enzyme-linked immunosorbent assay technique with the corresponding antibody in each kit. Absorbance readings of the samples were taken with a Victor X5 Microplate Reader (PerkinElmer, Waltham, MA, USA) set at 450 nm, and the concentrations were determined based on standard curves established with recombinant rat IL-1β and recombinant rat IL-10. Final results were given in pg/mL.

### 2.9. Histological Study

The jejunal tissue segments were fixed by immersion in a 10% formaldehyde solution via connection to a Minipuls^®^ 3 peristaltic pump (Gilson, Middleton, WI, USA), and the specimens were embedded in paraffin using a Leica ASP3005 tissue processor (Leica Biosystems, Nussloch, Germany). The tissues were then sectioned into 5 µm slices (four slices per animal) with a Leica RM2235 rotary microtome (Leica Biosystems, Nussloch, Germany), mounted on glass microscope slides, and stained with haematoxylin and eosin. Each histological section was divided into four quadrants, and one microscopic field per quadrant was randomly selected for analysis. To minimise spatial clustering and ensure a more representative sampling, care was taken to select fields that were not in close proximity to one another. A total of sixteen fields were analysed per animal. The sections were examined under 100× magnification with a Leica microscope, and microphotographs were used to measure villus length from the basal membrane to the tip of the microvilli. Results were expressed in millimetres (mm). Intestinal lesions were evaluated and classified according to five levels of ischemia/reperfusion (I/R) injury, following the scoring system of Chiu et al. [46]: Grade 0, normal mucosa; Grade 1, development of subepithelial space at the villus tip, often accompanied by oedema and vascular congestion; Grade 2, detachment of the epithelial layer from the lamina propria with moderate extension of the subepithelial space, villus tip fragmentation, and haemorrhage; Grade 3, partial loss of villus tips, extensive epithelial detachment, and fragmentation with the loss of the upper third of the villi; Grade 4, dilated and exposed capillaries with lost villi, though crypts remain present; Grade 5, haemorrhage, ulceration, disintegration of the lamina propria, and complete mucosal necrosis.

### 2.10. Statistical Analysis

Statistical analyses were conducted using SPSS Statistics^®^ 23.0 version. The Kruskal–Wallis test was used to assess the statistical significance of differences. For comparisons between two groups, the Mann-Whitney U test was applied. A significance level of *p* < 0.05 was considered. The Pearson chi-squared test was employed to analyse the severity of bowel tissue injury (*p* < 0.05). Data are presented as the mean ± standard deviation.

## 3. Results

### 3.1. Polyphenolic Content and Antioxidant Activity of F. vesiculosus Extract

In a previous study conducted by this research group, the optimisation of the extraction process of *Fucus vesiculosus* was carried out [44]. The results demonstrated that extraction times of 60 min and temperatures of 25 °C resulted in the *F. vesiculosus* extract with the highest antioxidant potential, obtaining a value of 89.74% with the 2,2-diphenyl-1-picrylhydrazyl free radical scavenging activity (DPPH-RSA) assay. The Folin–Ciocalteu method determined that this extract had a total phenolic content (TPC) of 13.61 mg phloroglucinol equivalents per 1 g of dry algae (PGE/g algae). In addition, the chromatographic characterisation of the extract by a reverse-phase high-performance liquid chromatography equipment coupled to a diode array detector (RP-HPLC-DAD) identified and quantified 10 polyphenols: floroglucinol (621.43 ± 7.28 μg/g), epicatechin (31.08 ± 1.47 μg/g), catechin (30.01 ± 1.95 μg/g), chlorogenic acid (20.27 ± 0.72 μg/g), rutin (17.10 ± 0.93 μg/g), gallic acid (15.26 ± 0.95 μg/g), vanillic acid (6.86 ± 0.07 μg/g), ferulic acid (6.07 ± 0.12 μg/g), caffeic acid (3.95 ± 0.14 μg/g) and coumaric acid (2.68 ± 0.07 μg/g). Among these, floroglucinol stood out due to its higher concentration compared to the others.

### 3.2. Effect of F. vesiculosus Treatment on Lipid Peroxidation: Determination of Malondialdehyde (MDA) Concentration in Intestinal Tissue

**Figure 1:**
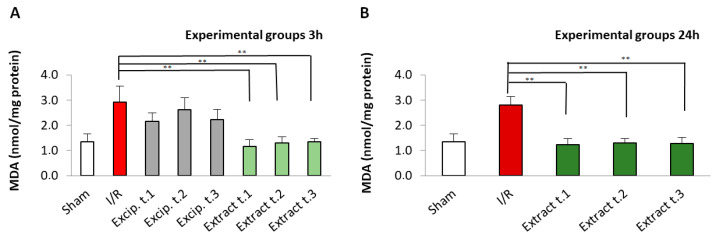
MDA levels present in the different experimental groups: (**A**) 60 min of ischemia and 3 h of reperfusion; (**B**) 60 min of ischemia and 24 h of reperfusion. Values expressed in nmol/mg protein (mean ± standard deviation). Significant differences between the treatment groups and the I/R group are indicated (** *p* < 0.01); Mann–Whitney U test, SPSS.

In Figure 1, an increase in lipid peroxidation damage was observed in the I/R group, in which MDA values increased, showing significant differences (*p* < 0.01) compared to the sham group. However, similar values to the sham control group were re-established in the groups treated with the algae extract. On the other hand, no significant differences were observed either in the different treatment times (t.1, t.2, t.3) or in the reperfusion times (3 and 24 h). The excipient-treated groups showed no significant differences compared to the I/R group.

### 3.3. Effect of F. vesiculosus Treatment on Superoxide Dismutase (SOD) Activity

**Figure 2:**
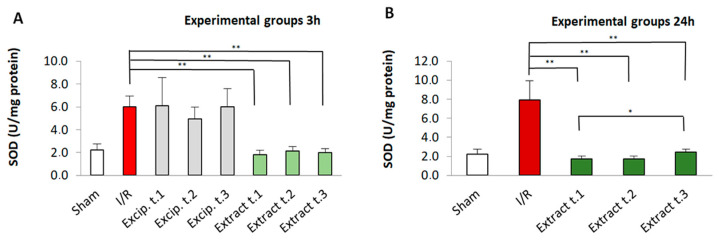
SOD levels present in the different experimental groups: (**A**) 60 min of ischemia and 3 h of reperfusion; (**B**) 60 min of ischemia and 24 h of reperfusion). Values expressed in U/mg protein (mean ± standard deviation. Significant differences between the treatment groups and the I/R group are indicated (* *p* < 0.05, ** *p* < 0.01); Mann–Whitney U test, SPSS.

The results presented in Figure 2 illustrates the impact of *F. vesiculosus* extract on SOD activity. When compared to the sham group, the level of intestinal SOD in the I/R group was significantly higher (*p* < 0.01). However, the enzyme level in the algae extract-treated group was significantly lower than in the I/R group (*p* < 0.01). Additionally, no significant differences were observed between the different administration times for groups with reperfusion periods of 3 h (Extract t.1, Extract t.2, and Extract t.3), while Extract t.1 showed significant differences with respect to the Extract t.3 group (*p* < 0.05) for groups subjected to 24 h of reperfusion. Furthermore, Extract t.3 (3 h reperfusion) differed significantly from Extract t.3 (24 h reperfusion). Lastly, no differences were found between the excipient-treated groups and the I/R group.

### 3.4. Effect of F. vesiculosus Treatment on Catalase (CAT) Activity

**Figure 3:**
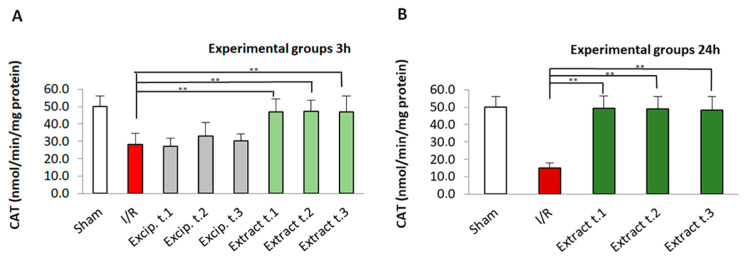
CAT levels present in the different experimental groups: (**A**) 60 min of ischemia and 3 h of reperfusion; (**B**) 60 min of ischemia and 24 h of reperfusion. Values expressed in nmol/min/mg protein (mean ± standard deviation). Significant differences between the treatment groups and the I/R group are indicated (** *p* < 0.01); Mann–Whitney U test, SPSS.

The effects of *F. vesiculosus* extract on CAT activity are shown in Figure 3. The activity of the antioxidant enzyme was significantly lower in the I/R group compared to the sham group and algae extract-treated groups (*p* < 0.01). In addition, no significant differences between the sham group and the algae extract-treated groups were shown. On the other hand, the excipient-treated groups showed no significant differences from the I/R group. In terms of the comparison between the different extract application times (Extract t.1, Extract t.2, and Extract t.3), as well as reperfusion times (3 and 24 h), no significant differences were observed for any of the groups compared.

### 3.5. Effect of F. vesiculosus Treatment on Glutathione Peroxidase (GPx) Activity

**Figure 4:**
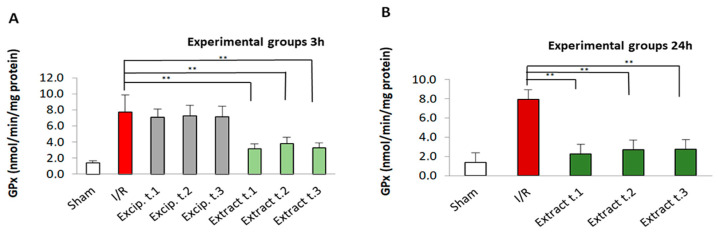
GPx levels present in the different experimental groups: (**A**) 60 min of ischemia and 3 h of reperfusion; (**B**) 60 min of ischemia and 24 h of reperfusion. Values expressed in nmol/min/mg protein (mean ± standard deviation. Significant differences between the treatment groups and the I/R group are indicated (** *p* < 0.01); Mann–Whitney U test, SPSS.

GPx enzyme activity increased significantly in the untreated I/R groups, whereas the algae extract-treated groups exhibited a significant reduction in GPx values following the I/R process (*p* < 0.01). On the other hand, the excipient-treated groups showed no significant differences compared to the I/R group. No significant differences were observed in the treatment in terms of time of application (Extract t.1, Extract t.2, and Extract t.3) for any of the reperfusion periods (3 and 24 h). These results can be seen in Figure 4. However, differences were observed between Extract t.1 (3 h reperfusion)/Extract t.1 (24 h reperfusion) and Extract t.2 (3 h reperfusion)/Extract t.2 (24 h reperfusion).

### 3.6. Effect of F. vesiculosus Treatment on Mieloperoxidase (MPO) Concentration

**Figure 5:**
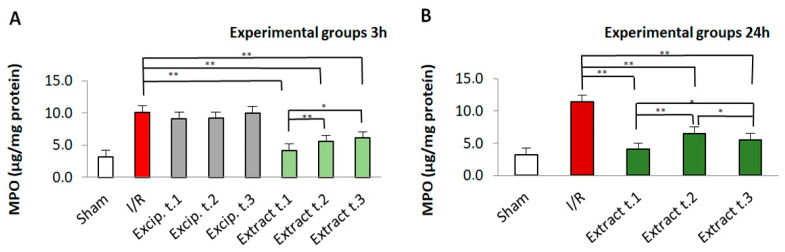
MPO levels present in the different experimental groups: (**A**) 60 min of ischemia and 3 h of reperfusion; (**B**) 60 min of ischemia and 24 h of reperfusion. Values expressed in µg/mg protein (mean ± standard deviation). Significant differences between the treatment groups and the I/R group are indicated (* *p* < 0.05, ** *p* < 0.01); Mann–Whitney U test, SPSS.

MPO values were significantly higher (*p* < 0.01) in the I/R group compared to the sham group, indicating oxidative damage. All groups treated with *F. vesiculosus* exhibited a significant reduction in MPO levels (*p* = 0.009). However, the excipient-treated groups did not show any significant decrease in MPO levels. Significant differences were also observed between the different application times of the seaweed extract. In this sense, *p*-values below 0.05 were obtained for the comparisons between Extract t.1/Extract t.2 and Extract t.1/Extract t.3 in rats reperfused for 3 h, as well as between the groups Extract t.1/Extract t.2, Extract t.1/Extract t.3 and Extract t.2/Extract t.3 in rats subjected to 24 h of reperfusion. These results are shown in Figure 5. The comparative analysis between the two reperfusion periods showed significant differences between Extract t.2 (3 h reperfusion)/Extract t.2 (24 h reperfusion).

### 3.7. Effect of F. vesiculosus Treatment on Inflammation Markers: Interleukins Il-1β and IL-10

**Figure 6:**
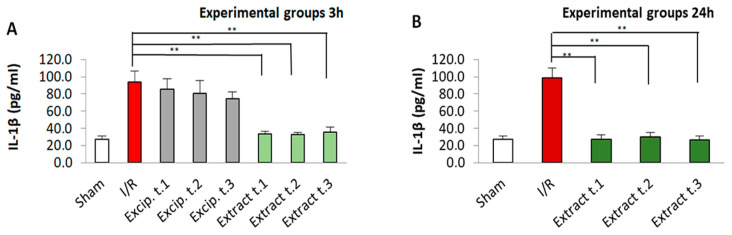
Il-1β levels present in the different experimental groups: (**A**) 60 min of ischemia and 3 h of reperfusion; (**B**) 60 min of ischemia and 24 h of reperfusion. Values expressed in µg/mg protein (mean ± standard deviation). Significant differences between the treatment groups and the I/R group are indicated (** *p* < 0.01); Mann–Whitney U test, SPSS.

**Figure 7:**
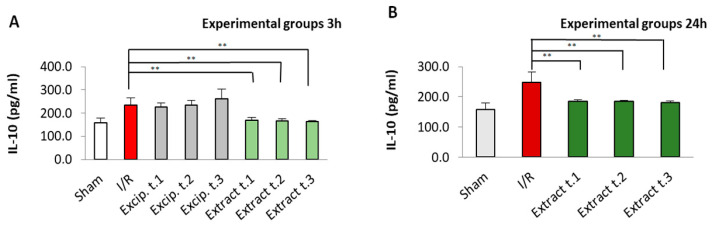
Il-10 levels present in the different experimental groups: (**A**) 60 min of ischemia and 3 h of reperfusion; (**B**) 60 min of ischemia and 24 h of reperfusion. Values expressed in µg/mg protein (mean ± standard deviation. Significant differences between the treatment groups and the I/R group are indicated (** *p* < 0.01); Mann–Whitney U test, SPSS.

The levels of interleukins, both Il-1β and Il-10, were significantly higher in the untreated I/R group compared to the sham group (*p* < 0.05). In contrast, the groups treated with the seaweed extract showed a significant reduction in their interleukin levels. For IL-10, values comparable to those of the sham group were restored in all treated groups, regardless of application time and reperfusion period. In the case of IL-1β, sham values were restored in all three groups (Extract t.1, t.2 and t.3) reperfused for 24 h, whereas this restoration was not observed in these groups after 3 h of reperfusion. No significant differences were observed for either Il-1β or Il-10 between the different treatment application times. All these results are presented in Figure 6 and Figure 7. However, when comparing the effect of the reperfusion period, differences in IL-1β levels were noted between the groups Extract t.3 (3 h reperfusion)/Extract t.3 (24 h reperfusion), while differences in IL-10 levels were observed in all three groups: Extract t.1 (3 h reperfusion)/Extract t.1 (24 h reperfusion), Extract t.2 (3 h reperfusion)/Extract t.2 (24 h reperfusion) and Extract t.3 (3 h reperfusion)/Extract t.3 (24 h reperfusion). The groups treated with the excipient showed no differences when compared to the I/R group.

The complete data for all biochemical parameters and their statistical analysis as well as the comparison between the two reperfusion times are provided in the Supplementary Materials (Tables S1 and S2).

### 3.8. Effects of F. vesiculosus Extract on Histological Alterations Induced by Intestinal I/R

**Figure 8:**
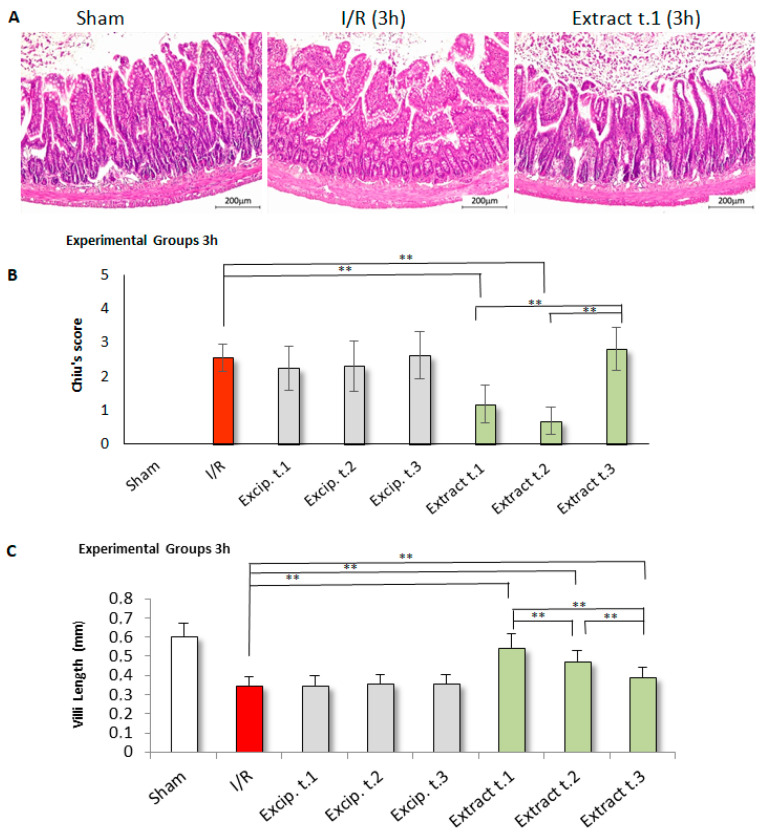
Histological results for the experimental groups after 3 h of reperfusion: (**A**) Tissue sections of small intestinal mucosa stained with H&E (100× magnification) revealed significant villus fragmentation and loss in the I/R group. In contrast, the villous structure was better preserved in the *F. vesiculosus* extract group. (**B**) Distribution of intestinal injury according to the Chiu scoring system (G0 = normal mucosa; G1 = development of subepithelial space at the villus tip, often accompanied by oedema and vascular congestion; G2 = detachment of the epithelial layer from the lamina propria with moderate extension of the subepithelial space, villus tip fragmentation, and haemorrhage; G3 = partial loss of villus tips, extensive epithelial detachment, and fragmentation with loss of the upper third of the villi; G4 = dilated and exposed capillaries with lost villi, though crypts remain present; G5 = haemorrhage, ulceration, disintegration of the lamina propria, and complete mucosal necrosis) indicated that the I/R group exhibited significant alterations, including fragmentation of the upper villus regions and loss of crypts (**C**) Furthermore, a marked reduction in villus length was noted in the I/R group compared to *F. vesiculosus* extracts with significant differences (** *p* < 0.01).

**Figure 9:**
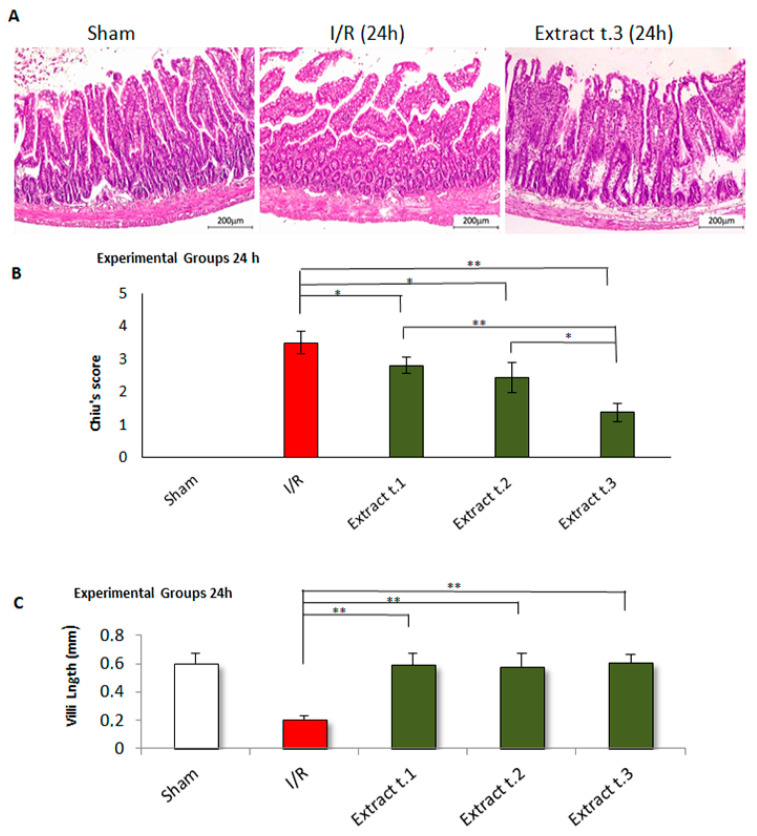
Histological results for the experimental groups after 24 h of reperfusion: (**A**) Tissue sections of small intestinal mucosa stained with H&E (100× magnification) revealed significant villus fragmentation and loss in the I/R group. In contrast, the villous structure was better preserved in the *F. vesiculosus* extract group. (**B**) Distribution of intestinal injury according to the Chiu scoring system (G0 = normal mucosa; G1 = development of subepithelial space at the villus tip, often accompanied by oedema and vascular congestion; G2 = detachment of the epithelial layer from the lamina propria with moderate extension of the subepithelial space, villus tip fragmentation, and haemorrhage; G3 = partial loss of villus tips, extensive epithelial detachment, and fragmentation with loss of the upper third of the villi; G4 = dilated and exposed capillaries with lost villi, though crypts remain present; G5 = haemorrhage, ulceration, disintegration of the lamina propria, and complete mucosal necrosis) indicated that the I/R group exhibited significant alterations, including fragmentation of the upper villus regions and loss of crypts (**C**) Furthermore, a marked reduction in villus length was noted in the I/R group compared to *F. vesiculosus* extracts with significant differences (* *p* < 0.05, ** *p* < 0.01).

Damage to the intestinal mucosa by I/R was assessed using the scoring scale outlined by Chiu et al. [46], with the results shown in Figure 8B and Figure 9B. In the sham group, histopathological examination revealed a typical mucosal structure (G0), characterised by well-organised, tall, and evenly spaced villi with consistent thickness. The crypts appeared tightly packed and contained a thin layer of lamina propria. In contrast, the I/R group displayed significant mucosal damage, with notable villus loss, atrophy, and fragmentation. Most sections were classified under grade G3, with some showing G4 damage. On the other hand, in the treated groups after 3 h of reperfusion (Figure 8B), the intestinal mucosa showed more favourable outcomes when the extract was administered either 24 h prior to ischemia (Extract t.1) or at the onset of ischemia (Extract t.2), with significant differences (*p* < 0.01) compared to the I/R group. In contrast, in the treated groups subjected to 24 h of reperfusion (Figure 9B), greater mucosal recovery was observed when the extract was administered at the beginning of the reperfusion phase (Extract t.3). Comparative analysis between the two reperfusion periods revealed significant differences in all three groups: Extract t.1 (3 h reperfusion) vs. Extract t.1 (24 h reperfusion) (*p* = 0.000), Extract t.2 (3 h reperfusion) vs. Extract t.2 (24 h reperfusion) (*p* = 0.001), and Extract t.3 (3 h reperfusion) vs. Extract t.3 (24 h reperfusion) (*p* = 0.001). No differences were observed in the groups treated with the excipient compared to the I/R group.

The villi in the groups treated with seaweed extract exhibited greater length and preservation compared to the untreated I/R group (Figure 8C and Figure 9C). Regarding the timing of administration, in the treated groups after 3 h of reperfusion (Figure 8C) significant differences were observed (*p* < 0.05) between groups, with the Extract t.1 showing a greater villus height, while no significant differences were observed between the treated groups after 24 h of reperfusion (Figure 9C). Comparison of the reperfusion times showed significant differences (*p* < 0.001) between Extract t.2 (3 h reperfusion) vs. Extract t.2 (24 h reperfusion) and Extract t.3 (3 h reperfusion) vs. Extract t.3 (24 h reperfusion). The groups treated with the excipient showed no differences when compared to the I/R group.

## 4. Discussion

Intestinal ischemia/reperfusion (I/R) injury is associated with numerous acute pathological conditions and plays a crucial role in several clinical and surgical procedures [13,47]. This pathophysiological process leads to a significant release of reactive oxygen species (ROS), which contribute to oxidative stress [2,6]. To combat ROS overproduction, organisms have developed a complex endogenous antioxidant defence system [1,48]. However, in the face of an overwhelming challenge, as may occur in the case of regional arterial occlusion, the provision of exogenous antioxidants helps ensure tissue survival [49].

Lipoperoxidation is a metabolic process where ROS cause the oxidative degradation of lipids, leading to the formation of malondialdehyde (MDA), a biomarker used to assess oxidative damage in tissues [50]. All intestinal samples treated with *F. vesiculosus* showed a significant decrease in MDA levels compared to the I/R group, suggesting that the antioxidants in the seaweed extract may inhibit ROS formation. Similar results have been reported in studies on other natural extracts, such as *Ganoderma lucidum* [51], *Phyllantus amarus* [12] and *Himathalia elongata* [52], where the extracts also reduced MDA levels, highlighting their potential to mitigate oxidative damage.

Antioxidant enzymes, including superoxide dismutase (SOD), catalase (CAT) and glutathione peroxidase (GPx), form the primary endogenous defence system against oxidative stress [53]. The main function of SOD is to convert the superoxide anion (O_2_^−^) into hydrogen peroxide (H_2_O_2_) and oxygen [54]. Then, CAT and GPx break down the H_2_O_2_ into water and oxygen [55]. An increase in SOD and GPx during mesenteric injury may indicate oxidative stress, with the cell possibly using this as an adaptive response to counter ROS production [56,57,58,59]. In this study, mesenteric obstruction significantly increased the levels of these enzymes compared to the sham group. However, the administration of the extract resulted in a reduction in enzyme activity, suggesting potential protective effects against tissue damage. These findings align with other studies, where increased SOD and GPx levels during I/R were associated with adaptive responses, and treatments with melatonin and thymoquinone [60], *Parquetina nigrescens* extract [59] and astaxanthin [61] reduced enzyme levels compared to the I/R group. On the other hand, CAT activity may decrease under oxidative stress, despite elevated ROS levels, possibly due to the presence of multiple isoenzymes with different functions. The assay used in our study measured total catalase activity without distinguishing between isoforms. But several studies have reported that CAT activity can be inversely correlated with oxidative damage [62,63,64]. In this context, the reduced CAT activity observed in the I/R group supports the presence of oxidative stress, whereas treatment with the algal extract significantly increased the enzyme levels. Previous studies evaluating the antioxidant potential of some polyphenols in experimental I/R models support our findings [65,66,67,68], showing a decrease in CAT activity in the I/R group, followed by a significant increase in enzyme activity after treatment with polyphenols. Moreover, since GPx and CAT use the same substrate, H_2_O_2_, the reduction in CAT activity could be leading to an increase in GPx activity as a compensatory mechanism, likewise the increased SOD levels could also be contributing to an accumulation of H_2_O_2_ [60].

Myeloperoxidase (MPO) is a lysosomal haemoprotein present in leukocytes and macrophages [69], and its activity is associated with inflammatory and oxidative stress processes [70]. MPO catalyses the conversion of chloride ions (Cl^−^) and H_2_O_2_ into hypochlorous acid (HClO), a potent oxidant that generates ROS [71,72]. In this study, the results reveal that *F. vesiculosus* treatment significantly reduces the increased MPO levels during the intestinal I/R process, suggesting that the algae extract is able to attenuate the oxidant–antioxidant imbalance during I/R. Other studies have also demonstrated that polyphenols present in natural products, such as quercetin, rutin, resveratrol, and ferric acid, can inhibit MPO activity, offering potential for reducing I/R injury [70].

One of the main responses of tissues when subjected to I/R is the inflammatory response, as the release of pro- and anti-inflammatory cytokines is directly related to the formation of ROS [73]. Tissues subjected to I/R experienced an increase in interleukin-1β (Il-1β) levels, which is justified by the increase in MPO, which promotes the infiltration of leukocytes and pro-inflammatory cytokines [12]. On the other hand, an increase in interleukin-10 (IL-10) expression was also observed. IL-10 is an anti-inflammatory cytokine that suppresses the expression of IL-1 β. However, several studies have found that both IL-1β and IL-10 increase after reperfusion in intestinal samples, rather than exhibiting an inverse relationship [74,75]. This could be interpreted as an adaptive mechanism by the organism to regulate the immune response in the damaged tissue [76]. Pretreatment with *F. vesiculosus* significantly attenuated the inflammatory response in the damaged tissues, reducing both IL-1β and IL-10 levels, suggesting that our treatment inhibits ROS production and attenuates the inflammatory response. These results are consistent with those of Ucar et al. [77], who also observed increases in both cytokines during intestinal I/R, and the administration of a treatment based on receptor blockade significantly decreased their expression.

Histopathological examination of jejunal tissue revealed that superior mesenteric artery occlusion for 60 min, followed by 3 and 24 h of reperfusion, caused severe damage to the intestinal mucosa. However, this damage was significantly reduced with treatment using *F. vesiculosus* extract. The injury grades, reflected in the Chiu score, were higher in the I/R group (grades 3–4) compared to the sham group (grade 0). In contrast, the treated samples showed a significant reduction in the score (grades 0–1–2). Additionally, the results demonstrated that the extract helped preserve the structure of the villi, with treated groups showing significant improvement in villus length compared to the I/R group.

The comparative study of treatment application times, in terms of degrees of injury, showed that in experimental groups subjected to 3 h of reperfusion, treatment at times t.1 and t.2 were most effective in reducing I/R injury. However, in groups subjected to 24 h of reperfusion, better results were observed when the extract was administered at the beginning of reperfusion (Extract t.3). Furthermore, t.1 and t.2 showed a greater reduction in damage with 3 h of reperfusion compared to the 24 h reperfusion period. However, t.3 showed better results after 24 h of reperfusion. These results suggest that when the treatment is administered before (t.1) or at the time of ischemia (t.2), it reduces oxidative injury, possibly due to its protective effect, as it proves effective even with just 3 h of reperfusion. On the other hand, when the extract is applied after ischemia (t.3), this protective effect is not observed, but a significant improvement in injury is noted after 24 h of reperfusion, suggesting it may act on ROS during this time interval. The comparative analysis of villus length also suggests that, for 3 h of reperfusion, Extract. t.1 yields better results, but Extract. t.3 works better with a long reperfusion period (24 h) than with a short one (3 h).

Overall, our results highlight not only the complexity of the mechanisms involved in intestinal ischemia/reperfusion injury but also the current limitations in understanding their temporal progression. The observed variability in the analysed parameters suggests that distinct pathological processes may be activated or suppressed at different time points during reperfusion. This highlights the need for future studies to more precisely define when and how oxidative, inflammatory and apoptotic mechanisms contribute to tissue damage in this model.

Finally, the groups that were treated with the excipient exhibited no significant differences when compared to the I/R group. This confirms that the observed effect is exclusively due to the extract, and the excipient shows no effect on the I/R injury.

## 5. Conclusions

In conclusion, our findings demonstrated that the *F. vesiculosus* extract holds significant potential in mitigating I/R injury in the intestines of rats. The extract effectively reduced lipid peroxidation, as evidenced by lower MDA levels, and helped preserve the antioxidant enzymatic balance by maintaining CAT, SOD, GPx and MPO activities. Furthermore, the treatment modulated the inflammatory response by reducing IL-1β and IL-10 cytokine levels towards normal levels. Histological analysis confirmed that the extract helped preserve intestinal mucosal architecture and reduce tissue damage. Nevertheless, the variability observed among the different extract administration times highlights the complexity of ischemia/reperfusion injury and underscores the need for further studies to better understand its temporal progression. Therefore, the use of *F. vesiculosus* extract appears promising and warrants further investigation as a potential treatment for preventing ischemic processes in mesenteric thromboembolism and during surgical procedures.

## Figures and Tables

**Table 1:** Experimental Groups.

Groups		N	Treatment/Excipient	Ischemia	Reperfusion
Control	SHAM:	6	-	-	-
	I/R 3H	6	-	60 min	3 h
	I/R 24H	6	-	60 min	24 h
Excipient	Excip. t.1	6	0.5 mL of 10% ethanol	60 min	3 h
	Excip t.2	6	0.5 mL of 10% ethanol	60 min	3 h
	Excip t.3	6	0.5 mL of 10% ethanol	60 min	3 h
Algae Extract	Extract t.1	6	0.5 mL of extract (222 mg/kg)	60 min	3 h
	Extract t.2	6	0.5 mL of extract (222 mg/kg)	60 min	3 h
	Extract t.3	6	0.5 mL of extract (222 mg/kg)	60 min	3 h
	Extract t.1	6	0.5 mL of extract (222 mg/kg)	60 min	24 h
	Extract t.2	6	0.5 mL of extract (222 mg/kg)	60 min	24 h
	Extract t.3	6	0.5 mL of extract (222 mg/kg)	60 min	24 h

t.1: Treatment administered 24 h before surgery. t.2: Treatment administered at the initiation of ischemia. t.3: Treatment administered at the time of initiation of reperfusion.

## Data Availability

It is not possible to publicly share the research data because the data are from the doctoral thesis of the researcher Desiree Sánchez-Bonet. However, after the defence of her work, access to the data can be requested by email to: desanbo@gmail.com.

## Supplementary Materials

The following supporting information can be downloaded at: https://www.mdpi.com/article/10.3390/antiox14060624/s1, Table S1: Analytical parameter results. Statistical comparison between treatments and I/R or Sham groups. Table S2: Analytical parameter results. Statistical comparison between treatments at different reperfusion times.
