# Supplementary material for: Antioxidant Effect of a Fucus vesiculosus Extract on Intestinal Ischemia/Reperfusion Injury in Rats: A Biochemical and Histological Study
Source: Antioxidants (Basel). 2025 May 23;14(6):624. doi: 10.3390/antiox14060624 (PMC12189128; doi:10.3390/antiox14060624)
Supplement: Supplementary file 1 [file antioxidants-14-00624-s001.zip › antioxidants-3597993-supplementary.pdf]

Table S.1_ Analytical parameter results. Statistical comparison between treatments and I/R or Sham groups.

			REPERFUSION 3H			REPERFUSION 24H		
Lipoperoxidation	MDA (nmol/mg protein)	**Sham**	1.35 ± 0.32			1.35 ± 0.32		
		**I/R**	2.91 ± 0.65			2.79 ± 0.35		
			**t.1**	**t.2**	**t.3**	**t.1**	**t.2**	**t.3**
		Excip.	2.16 ± 0.40	2.61 ± 0.48	2.18 ± 0.42			
		Extract	1.17 ± 0.26**	1.29 ± 0.24**	1.33 ± 0.14**	1.22 ± 0.26**	1.29 ± 0.17**	1.27 ± 0.25**
Enzyme Activity in Oxidative Stress	SOD (u/mg protein)	Sham	2.22 ± 0.54			2.22 ± 0.54		
		I/R	6.02 ± 0.91			7.93 ± 1.98		
			**t.1**	**t.2**	**t.3**	**t.1**	**t.2**	**t.3**
		Excip.	6.11 ± 2.46	4.96 ± 1.02	6.02 ± 1.55			
		Extract	1.80 ± 0.40**	2.15 ± 0.36**	1.98 ± 0.37**	1.71 ± 0.34**	1.72 ± 0.33**	2.44 ± 0.30**
	CAT (nmol/min/mg protein)	Sham	49.96 ± 6.15			49.96 ± 6.15		
		I/R	28.30 ± 6.39			15.06 ± 2.79		
			**t.1**	**t.2**	**t.3**	**t.1**	**t.2**	**t.3**
		Excip.	27.09 ± 4.57	33.09 ± 7.84	30.13± 3.98			
		Extract	46.98 ± 7.25*	47.09 ± 6.50*	46.72 ± 9.51*	49.39 ± 6.88**	49.04 ± 6.96**	48.41 ± 7.68**
	GPx (nmol/min/mg protein)	Sham	1.39 ± 0.27			1.39 ± 0.27		
		I/R	7.71 ± 2.14			7.92 ± 2.40		
			**t.1**	**t.2**	**t.3**	**t.1**	**t.2**	**t.3**
		Excip.	7.06 ± 1.04	7.25 ± 1.34	7.13 ± 1.32			
		Extract	3.14 ± 0.63**††	3.81 ± 0.75**††	3.26 ± 0.62**††	2.27 ± 0.32**††	2.69 ± 0.36**††	2.73 ± 0.46**††
	MPO (ng/mg protein)	Sham	3.81 ± 0.42			3.81 ± 0.42		
		I/R	10.08 ± 0.98			11.42 ± 1.46		
			**t.1**	**t.2**	**t.3**	**t.1**	**t.2**	**t.3**
		Excip.	9.11 ± 1.42	9.16 ± 1.73	9.99 ± 0.72			
		Extract	4.19 ± 0.57**††	5.57 ± 0.30**††	6.07 ± 1.30**††	4.02 ± 0.61**††	6.51 ± 0.57**††	5.49 ± 0.51**††
Inflammation	IL-1β (pg/ml)	Sham	27.32 ± 3.58			27.32 ± 3.58		
		I/R	93.80 ± 12.79			98.86 ± 11.39		
			**t.1**	**t.2**	**t.3**	**t.1**	**t.2**	**t.3**
		Excip.	85.67 ± 11.93	80.98 ± 14.45	74.75 ± 7.35			
		Extract	33.57±3.35**†	32.69±2.76**†	35.31±6.06**†	27.56 ± 4.72**	30.12 ± 5.25**	26.79 ± 3.94**
	IL-10 (pg/ml)	Sham	158.16 ± 21.10			158.16 ± 21.10		
		I/R	233.27 ± 32.49			247.32 ± 36.21		
			**t.1**	**t.2**	**t.3**	**t.1**	**t.2**	**t.3**
		Excip.	226.63±17.78	235.22±19.52	261.35±43.14			
		Extract	185.63±5.32*	184.88±3.69*	180.16±6.70**	169.16±11.50**	166.88±9.77**	164.04±3.35**

*(p < 0.05) **(p < 0 .01) significant differences with respect to I/R group.
†(p < 0.05) †† (p < 0 .01) significant differences with respect to sham group.
Mean ± standard deviation (n = 6).

Table S.2_ Analytical parameter results. Statistical comparison between treatments at different reperfusion times

	T.1 3H *vs.* T.1 24H	T.2 3H *vs.* T.2 24H	T.3 3H *vs.* T.3 24H
**MDA** (nmol/mg protein)	1.17 ± 0.26/ 1.22 ± 0.26	1.29 ± 0.24/ 1.29 ± 0.17	1.33 ± 0.14/ 1.27 ± 0.25
	p-value=0.602	p-value =0.754	p-value =0.754
**SOD** (u/mg protein)	1.80 ± 0.40/ 1.71 ± 0.34	2.15 ± 0.36/ 1.72 ± 0.33	1.98 ±0.37/ 2.44 ± 0.30
	p-value =0.754	p-value =0.465	**p-value=0.047***
**CAT** (nmol/min/mg protein)	46.98 ± 7.20/ 49.39 ± 6.90	47.09 ± 6.50/ 49.04 ± 7.00	46.72 ± 9.50/ 48.41 ± 7.70
	p-value=0.602	p-value=0.602	p-value=0.754
**GPx** (nmol/min/mg protein)	3.14 ± 0.63/ 2.27 ± 0.33	3.81 ± 0.75/ 2.69 ± 0.36	3.26 ± 0.62/ 2.73 ± 0.55
	**p-value=0.028***	**p-value=0.028***	p-value=0.251
**MPO** (ng/mg protein)	4.19 ± 0.57/ 4.02 ± 0.61	5.57 ± 0.30/ 6.51 ± 0.57	6.07 ± 1.30/ 5.49 ± 0.51
	p-value=0.754	**p-value=0.016***	p-value=0.175
**IL-1β** (pg/ml)	33.57 ± 3.35/ 27.56 ± 4.72	32.69 ± 2.76/ 30.12 ± 5.25	35.31 ± 6.06/ 26.79 ± 3.94
	p-value=0.175	p-value=0.347	**p-value=0.009****
**IL-10** (pg/ml)	185.60 ± 5.30/ 169.20 ± 11.50	184.90 ± 3.70/ 166.90 ± 9.80	180.20 ± 6.70/ 164.04 ± 3.30
	**p-value=0.009****	**p-value=0.028***	**p-value=0.009****

Mean ± standard deviation (n = 6).

p < 0.05, **p < 0.01 significant differences between groups.
